# Platinum-Taxol non-cross resistance in epithelial ovarian cancer.

**DOI:** 10.1038/bjc.1995.253

**Published:** 1995-06

**Authors:** M. E. Gore, N. Preston, R. P. A'Hern, C. Hill, P. Mitchell, J. Chang, M. Nicolson

**Affiliations:** Gynaecology Unit, Royal Marsden Hospital, London, UK.

## Abstract

The aim of this study was to assess the clinical evidence for platinum-Taxol non-cross-resistance in patients with epithelial ovarian cancer. Unlike other studies, only patients who had demonstrably progressive disease on platinum therapy were analysed. Patients received 135-200 mg m-2 of Taxol over 3 or 24 h and all patients were assessed for response by computerised axial tomography. The overall response rate was 22.2% (8/36 patients, 95% CI 10-39%). Only patients who received > or = 175 mg m-2 of Taxol responded (26.7%; 8/30 patients, 95% CI 12-46%). No complete responses were seen and the duration of response was short, median 7 months (range 5-9+). Response was associated with a short treatment-free interval (P = 0.02); only those who were treated immediately after they had progressed on their previous platinum therapy responded. Response duration was associated with a good performance status (P < 0.05). Platinum and Taxol are non-cross-resistant in a proportion of patients and therefore patients who are resistant to platinum compounds may benefit from Taxol although the duration of any response is short. These data support current strategies that involve combining Taxol with platinum compounds as first-line therapy in advanced epithelial ovarian cancer.


© 1995 Stockton Press Ltd All rights reserved 0007-0920/95 $12.00

### Platinum–Taxol non-cross resistance in epithelial ovarian cancer

ME Gore, N Preston, RP A'Hern, C Hill, P Mitchell, J Chang and M Nicolson

*Gynaecology Unit, The Royal Marsden Hospital, Fulham Road, London SW3 6JJ, UK.*

**Summary** The aim of this study was to assess the clinical evidence for platinum–Taxol non-cross-resistance in patients with epithelial ovarian cancer. Unlike other studies, only patients who had demonstrably progressive disease on platinum therapy were analysed. Patients received 135–200 mg m$^{-2}$ of Taxol over 3 or 24 h and all patients were assessed for response by computerised axial tomography. The overall response rate was 22.2% (8 36 patients, 95% CI 10–39%). Only patients who received ⩾ 175 mg m$^{-2}$ of Taxol responded (26.7%; 8 30 patients, 95% CI 12–46%). No complete responses were seen and the duration of response was short, median 7 months (range 5–9+). Response was associated with a short treatment-free interval ($P$=0.02); only those who were treated immediately after they had progressed on their previous platinum therapy responded. Response duration was associated with a good performance status ($P$<0.05). Platinum and Taxol are non-cross-resistant in a proportion of patients and therefore patients who are resistant to platinum compounds may benefit from Taxol although the duration of any response is short. These data support current strategies that involve combining Taxol with platinum compounds as first-line therapy in advanced epithelial ovarian cancer.

**Keywords:** epithelial ovarian cancer; Taxol; platinum; cross-resistance

Taxol was first isolated from the bark of the Pacific yew (*Taxus brevifolia*) in 1971 (Wani *et al.*, 1971) and has since been shown to have a wide spectrum of biological activity (Slichenmeyer and Von Hoff, 1991) but its main antineoplastic effect appears to be the promotion of polymerisation of microtubules, resulting in the formation of stable non-functional microtubules (Schiff *et al.*, 1979). The drug is active in patients with relapsed epithelial ovarian cancer, and cumulative data suggest that the response rate in this group of patients is 13–50% (McGuire *et al.*, 1989; Thigpen *et al.*, 1990; Enzig *et al.*, 1992; Sarosy *et al.*, 1992; Swenerton *et al.*, 1993). However, one of the most important considerations in the design of phase II studies in relapsed ovarian cancer is patient selection with regard to previous treatment. It is now well established that patients are more likely to respond to second-line chemotherapy the longer the time interval between the end of treatment and their relapse (Blackledge *et al.*, 1989; Gore *et al.*, 1990; Markman *et al.*, 1991). The importance of patient selection is even greater when evaluating whether or not a new compound is cross-resistant to standard therapy. Trimble *et al.* (1993) have demonstrated that Taxol is active in patients with poor-prognosis relapsed epithelial ovarian cancer. Their study included patients who had responded to platinum-based chemotherapy and relapsed within 3 months as well as patients with truly platinum-refractory disease, i.e. progressing while on platinum. They did not, however, address the issue of Taxol–platinum non-cross-resistance and its true incidence has not been studied. Non-cross-resistance can only be confidently reported in a population of patients that have truly platinum-refractory disease and that can only be definitely assessed in the presence of measurable progressive disease while the patient is receiving platinum-based chemotherapy. We present here our experience of Taxol given to a group of patients that have truly platinum-refractory disease and thus report the clinical evidence for, and incidence of, Taxol–platinum non-cross-resistance.

### Patients and methods

### *Patients*

Between October 1991 and October 1993 75 patients with refractory or relapsed epithelial ovarian cancer were entered into three consecutive multicentre trials of Taxol (015, 052, 005, Bristol-Myers Squibb). Thirty-six of these patients had progressive disease as defined by UICC criteria while on platinum therapy. These patients were therefore truly platinum resistant and included in the analysis.

The median age of the patients was 55 years (range 27–72) and their ECOG performance status was 0 (six patients), 1 (23 patients) or 2 (seven patients). Resistance to platinum was documented by computerised axial tomography in 27 patients, by second-look laparotomy in four patients and by clinical examination in conjunction with serum CA125 estimation in five patients. Patient characteristics and details of previous treatments are set out in Tables I and II.

Patients could only enter these studies if they had histologically proven epithelial ovarian cancer, ECOG status of 0–2, absolute granulocyte count ⩽ $1.5 \times 10^9 l^{-1}$, platelet count ⩾ $100 \times 10^9 l^{-1}$, bilirubin < 2 × upper limit of normal and creatinine ⩽ 2 × upper limit of normal. Patients were excluded if they had serious, inadequately controlled cardiac disease, pre-existing peripheral neuropathy > grade 2 or were known to have an allergic reaction to drugs containing Cremophor. All patients gave written informed consent according to guidelines laid down by the Royal Marsden Hospital Ethics Committee.

### *Treatment*

Patients received Taxol 135 mg m$^{-2}$ (six patients), 175 mg m$^{-2}$ (24 patients) or 200 mg m$^{-2}$ (six patients) intravenously over 3 h (33 patients) or 24 h (three patients). Taxol was administered from a glass bottle through non-PVC lines with an in-line filter attached. Patients were premedicated before Taxol administration as follows: 20 mg of dexamethasone orally 12 and 6 h before Taxol and cimetidine 300 mg with chlorpheniramine 10 mg intravenously 30 min before Taxol administration. Taxol was administered every 21 days.

### *Response assessment*

Patients were assessed for response after every 2–3 cycles by computerised axial tomography. Serum CA125 concentration was not used for response assessment. Response was defined according to UICC criteria: complete response (CR), complete disappearance of all disease for at least 4 weeks; partial response (PR), a decrease of 50% in the sum of the products of the perpendicular diameter of all measured lesions without the appearance of any new lesions for at least 4 weeks;

Correspondence: ME Gore
Received 12 September 1994; revised 1 December 1994; accepted 4 January 1995

**Table I** Patient characteristics

| | *No. of patients* |
|---|---|
| Taxol | |
| 135 mg m$^{-2}$ | 6 |
| 175 mg m$^{-2}$ | 24 |
| 200 mg m$^{-2}$ | 6 |
| Age | |
| $\geqslant$ 55 | 16 |
| $<$ 55 | 20 |
| Histology | |
| Serous | 24 |
| Non-serous | 12 |
| Performance status | |
| 0 | 6 |
| 1 | 23 |
| 2 | 7 |
| Haemoglobin (g 100 ml$^{-1}$) | |
| $>$ 11 | 24 |
| 9.5 | 10 |
| 8–9.4 | 2 |
| Treatment-free interval (months) | |
| $\geqslant$ 2 | 20 |
| $<$ 2 | 16 |
| Number of tumour sites | |
| $\geqslant$ 3 | 22 |
| $<$ 3 | 14 |
| Maximum tumour diameter (cm) | |
| $\geqslant$ 5 | 24 |
| $<$ 5 | 12 |

progressive disease (PD), development of new lesions or an increase in any measured lesion of $\geqslant$ 25% of the products of two perpendicular diameters; stable disease (SD), no change in measurable lesions or changes that do not fulfil the criteria for either PR or PD for at least 8 weeks.

### *Statistical methods*

Exact confidence intervals on response rates are quoted. Duration of response was measured from the date of the first treatment to the date progression and was confirmed by computerised axial tomography. We examined a number of patient characteristics to assess their role as predictors of response and response duration using log-rank analysis (Taxol dose, histology, age, performance status, haemoglobin, treatment-free interval, number of disease sites, size of largest deposit).

### Results

The overall response rate was 22.2% (8/36, 95% CI 10–39%) but no patient responded to 135 mg m$^{-2}$ Taxol (0/6) whereas 26.7% (8/30, 95% CI 12–46%) patients responded to $\geqslant$ 175 mg m$^{-2}$ Taxol. All patients received 1–10 cycles (median 3) of Taxol, with responders receiving 6–10 cycles (median 8). The median duration of response was 7 months (range 5–9 + ). All but three patients relapsed while on treatment; two patients developed disease progression 2 and 3 months after stopping Taxol and one patient who has just completed treatment remains in PR at 9 + months. There were no responses in the six patients who were resistant to combination platinum-based chemotherapy or in the six patients who had previously received $\geqslant$ 3 prior treatments. Seven patients had stabilisation of their disease lasting 1–12 + months (median 4). The median survival for all 36 patients analysed was 26 weeks (range 2–117). Response was associated with the treatment-free interval; all responders were in the group of patients treated immediately after progressing on platinum therapy (8/20, $P = 0.02$). There was also

**Table II** Treatment characteristics of 36 patients with platinum-resistant disease treated with Taxol

| | *No. of patients* |
|---|---|
| Prior treatment | |
| 1 | 12 |
| 2 | 18 |
| $\geqslant$ 3 | 6 |
| Resistant to | |
| Single-agent platinum | 30 |
| Combination platinum therapy | 6 |
| Carboplatin | 24 |
| Cisplatin | 6 |
| Carboplatin and cisplatin | 6 |

a trend for response to be associated with Taxol dose (0/6 responders at 135 mg m$^{-2}$, 6/24 responders at 175 mg m$^{-2}$, 2/6 responders at 200 mg m$^{-2}$, $P = 0.06$). A longer duration of response, as measured by the progression-free interval, was associated with a good performance status ($P = <0.05$) and there was a trend for patients with anaemia to have a shorter progression-free interval ($P = 0.07$).

### Discussion

Clinical evidence of non-cross-resistance between chemotherapeutic agents is important to establish in order to design more effective regimens and to help validate *in vitro* chemosensitivity assays. Phase II studies can produce misleading data in this area owing to patient selection. This is particularly true of epithelial ovarian cancer, of which the treatment-free interval is a powerful predictor of response in patients who have relapsed. For instance, patients who relapse at a disease-free interval of 1–2 years have a 27–33% chance of responding to a rechallenge with platinum-based chemotherapy, whereas response rates are 57–75% for patients who relapse after 2 years (Gore *et al.*, 1990; Markman *et al.*, 1991). A recent review of the phase II studies of Taxol in epithelial ovarian cancer has shown that a similar treatment-free interval–response relationship probably also exists for Taxol (Hansen *et al.*, 1993). Trimble *et al.* (1993) have shown that Taxol is active in a particularly poor-prognosis group of patients, that is those with progressive disease on treatment or early relapse within 3 months of their previous therapy. However, that study does not adequately address the issue of cross-resistance between platinum compounds and Taxol because of the inclusion of this latter group of patients. In order to assess reliably the frequency of Taxol–platinum non-cross-resistance, it is necessary to assess the efficacy of Taxol in patients who have progressive disease while on platinum. Patients who had no change in tumour measurements were not analysed in this study because the objective measurement of disease in epithelial ovarian cancer is notoriously inaccurate and we wanted to be absolutely sure that the patients being assessed for Taxol–platinum non-cross-resistance were truly platinum refractory.

Our study demonstrates that Taxol and platinum are non-cross-resistant in 26.7% of patients when doses of $\geqslant$ 175 mg m$^{-2}$ Taxol are used. These data suggest that it is logical to combine Taxol with platinum compounds as first-line therapy for treatment of epithelial ovarian cancer. However, the development of Taxol resistance appears surprisingly quickly. All but two of the responders relapsed while on treatment and the median duration of response was only 7 months (range 5–9 + ). The two patients who relapsed off treatment did so at 2 and 3 months after stopping Taxol. A careful assessment of the symptomatic benefit that is likely to be derived from using Taxol in a platinum-refractory patient needs to be weighed against cost and toxicity.

All our patients were resistant to carboplatin and therefore our data mainly relate to carboplatin–Taxol cross-resistance.

However, we have previously shown that there is cross-resistance between carboplatin and cisplatin in most patients (Gore *et al*., 1989), and we can therefore extrapolate our data to suggest that it is likely that Taxol and cisplatin are non-cross-resistant in at least a proportion of patients. These data support the current strategies exploring combinations of Taxol and platinum compounds as first-line therapy for advanced epithelial ovarian cancer.

### References

BLACKLEDGE G, LAWTON R, REDMAN C AND KELLY K. (1989) Response of patients in phase II studies of chemotherapy in ovarian cancer: implications for patient treatment and the design of phase II trials. *Br. J. Cancer*, **59**, 650–653.

EINZIG AI, WIERNIK PH, SASLOFF J, RUNOWICZ DD AND GOLDBER GL. (1992). Phase II study and long-term follow up of patients treated with taxol for advanced ovarian adenocarcinoma. *J. Clin. Oncol.*, **10**, 1748–1753.

GORE ME, FRYATT I, WILTSHAW E, DAWSON T, ROBINSON BA AND CALVERT AH. (1989). Cisplatin/carboplatin cross-resistance in ovarian cancer. *Br. J. Cancer*, **60**, 751–754.

GORE ME, FRYATT I, WILTSHAW E AND DAWSON T. (1990). Treatment of relapsed carcinoma of the ovary with cisplatin or carboplatin following initial treatment with these compounds. *Gynecol. Oncol.*, **36**, 207–211.

HANSEN HH, EISENHAUER EA, HANSEN M, NEIJI JP, PICCART MJ, SESSA C AND THIGPEN JT. (1993). New cytostatic drugs in ovarian cancer. *Ann. Oncol.*, **4**, S63–S70.

MARKMAN M, ROTHMAN R, HAKES T, REICHMAN B, HOSKINS W, RUBIN S, JONES W, ALMADRONES L AND LEWIS JL. (1991). Second-line platinum therapy in patients with ovarian cancer previously treated with cisplatin. *J. Clin. Oncol.*, **9**, 389–393.

McGUIRE WP, ROWINSKY EK, ROSENSHEIN NB, GRAMBINE FC, ETTINGER DS, ARMSTRONG KD AND DOMEHOUSER RC. (1989). Taxol: a unique antineoplastic agent with significant activity in advanced ovarian epithelial neoplasms. *Ann. Intern. Med.*, **111**, 273–279.

SAROSY G, KOHN E, LINK C, ADAMO D, DAVIS P, OGNIBENE F, GODSPIEL B, CHRISTIAN M AND REED E. (1992). Taxol dose intensification (DI) in patients with recurrent ovarian cancer. *Proc. Am. Soc. Clin. Oncol.*, **11**, 226.

SCHIFF PB, FANT J AND HORWITZ SB. (1979). Promotion of microtubule assembly *in vitro* by taxol. *Nature*, **277**, 665–667.

SLICHENMEYER WJ AND VON HOFF DD. (1991). Taxol: a new and effective anticancer drug. *Anti-Cancer Drugs*, **2**, 519–530.

SWENERTON K, EISENHAUER E, TEN BOKKEL HUININK W, MYLES J, MANGIONI C, VAN DER BURG M, KERR I, GIANNI L, VERMORKEN J, BURSER K, SADURA A, BACON M, SANTABARBARA P, ONETTO N AND CANETTA R. (1993). Taxol in relapsed ovarian cancer: high *vs* low dose and short *vs* long infusion: a European–Canadian study coordinated by the NCI Canada Clinical Trials Group. *Proc. Am. Soc. Clin. Oncol.*, **12**, 810.

THIGPEN T, BLESSING J, BALL H, HUMMEL S AND BARRET R. (1990). Phase II trial of taxol as a second-line therapy for ovarian carcinoma: Gynaecologic Oncology Group Study. *Proc. Am. Soc. Clin. Oncol.*, **9**, 604.

TRIMBLE EL, ADAMS JD, VENA D, HAWKINS MJ, FRIEDMAN MA, FISHERMAN JS, CHRISTIAN MC, CANETTA R, ONETTO N, HAYN R AND ARBUCK SG. (1993). Paclitaxel for platinum-refractory ovarian cancer: results from the first 1,000 patients registered to National Cancer Treatment Referral Centre 9103. *J. Clin. Oncol.*, **11**, 2405–2410.

WANI MC, TAYLOR HL, WALL ME, COGGON P AND McPHAIL AT. (1971). Plant antitumour agents VI. The isolation and structure of taxol, a novel antileukemic and antitumour agent from *Taxus brevifolia*. *J. Am. Chem. Soc.*, **93**, 2325–2329.


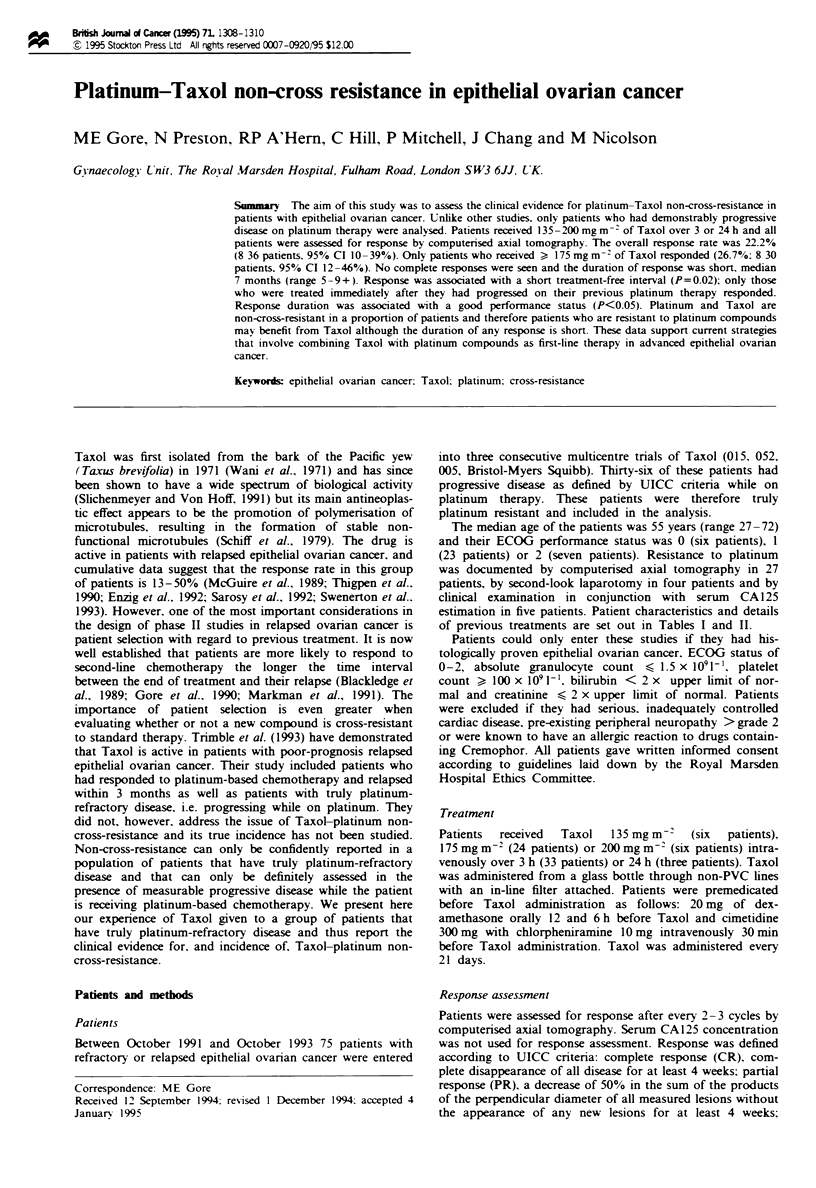

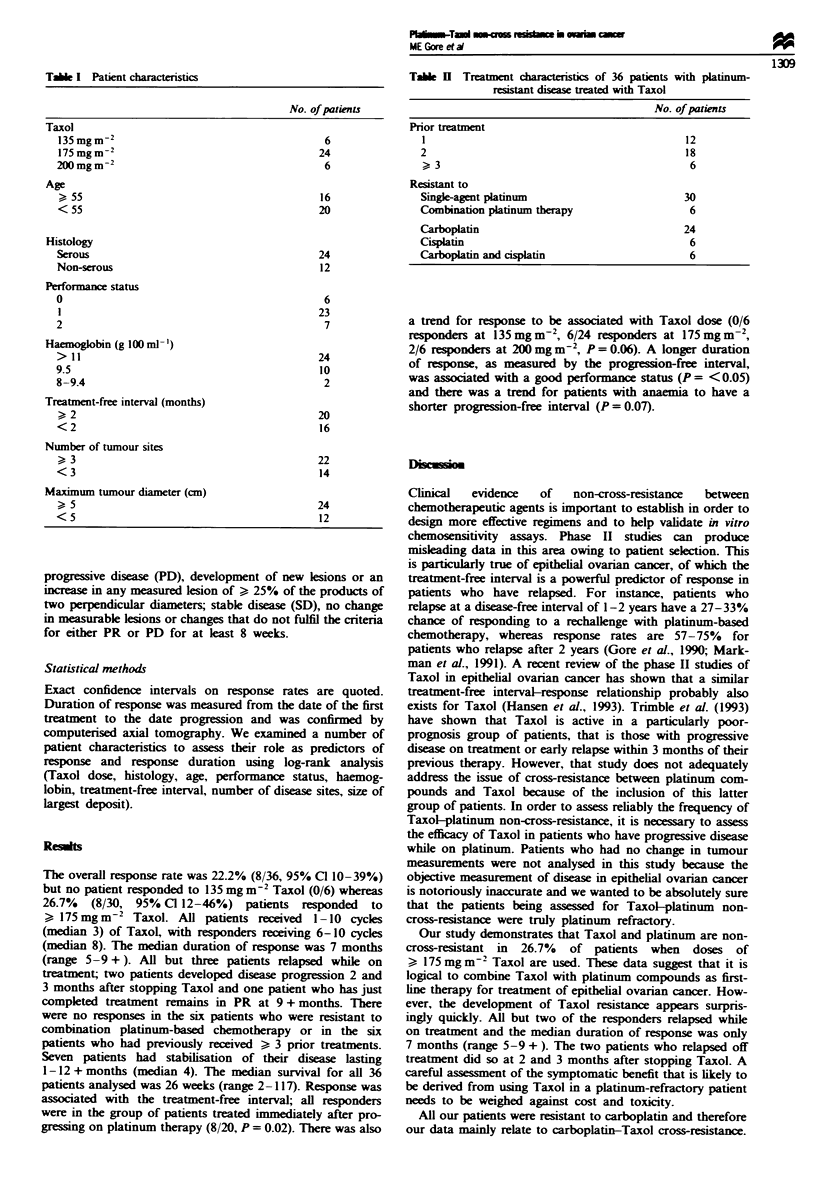

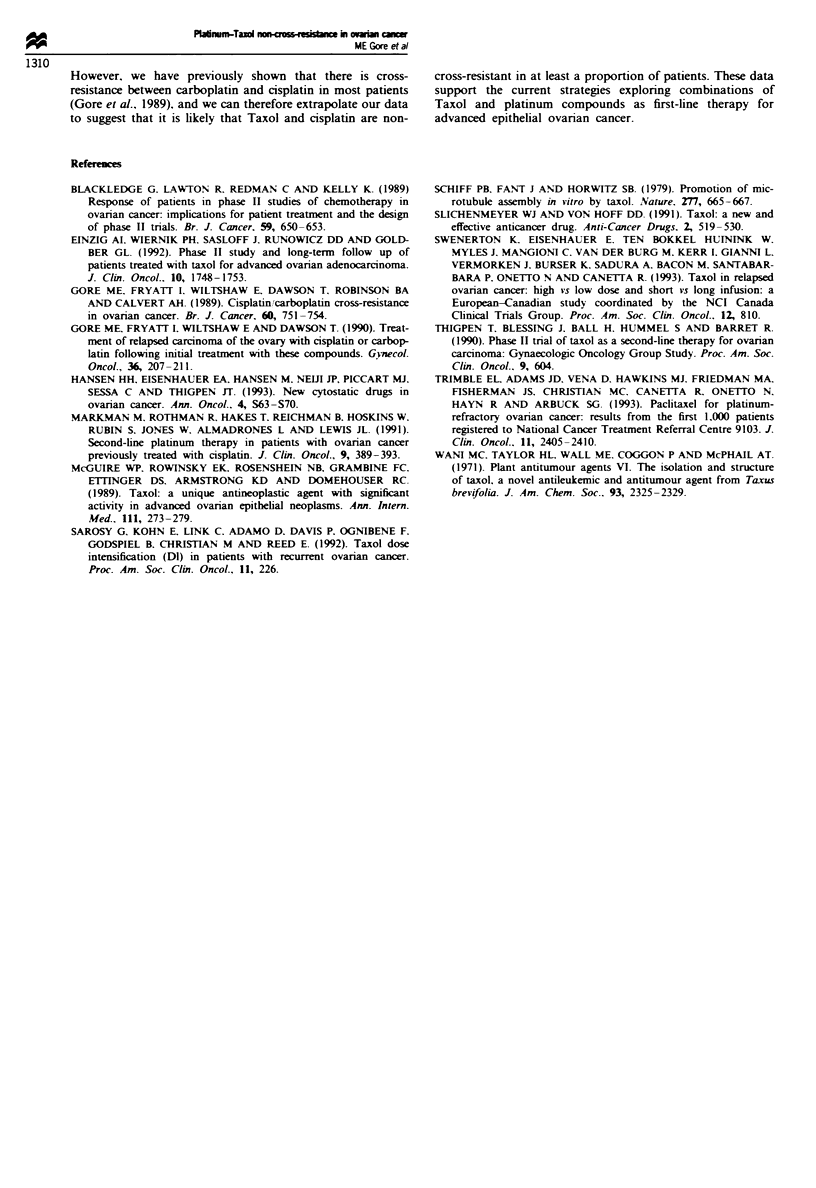


## References

[OCR_00344] Blackledge G., Lawton F., Redman C., Kelly K. (1989). Response of patients in phase II studies of chemotherapy in ovarian cancer: implications for patient treatment and the design of phase II trials.. Br J Cancer.

[OCR_00351] Einzig A. I., Wiernik P. H., Sasloff J., Runowicz C. D., Goldberg G. L. (1992). Phase II study and long-term follow-up of patients treated with taxol for advanced ovarian adenocarcinoma.. J Clin Oncol.

[OCR_00361] Gore M. E., Fryatt I., Wiltshaw E., Dawson T. (1990). Treatment of relapsed carcinoma of the ovary with cisplatin or carboplatin following initial treatment with these compounds.. Gynecol Oncol.

[OCR_00373] Markman M., Rothman R., Hakes T., Reichman B., Hoskins W., Rubin S., Jones W., Almadrones L., Lewis J. L. (1991). Second-line platinum therapy in patients with ovarian cancer previously treated with cisplatin.. J Clin Oncol.

[OCR_00379] McGuire W. P., Rowinsky E. K., Rosenshein N. B., Grumbine F. C., Ettinger D. S., Armstrong D. K., Donehower R. C. (1989). Taxol: a unique antineoplastic agent with significant activity in advanced ovarian epithelial neoplasms.. Ann Intern Med.

[OCR_00391] Schiff P. B., Fant J., Horwitz S. B. (1979). Promotion of microtubule assembly in vitro by taxol.. Nature.

[OCR_00395] Slichenmyer W. J., Von Hoff D. D. (1991). Taxol: a new and effective anti-cancer drug.. Anticancer Drugs.

[OCR_00412] Trimble E. L., Adams J. D., Vena D., Hawkins M. J., Friedman M. A., Fisherman J. S., Christian M. C., Canetta R., Onetto N., Hayn R. (1993). Paclitaxel for platinum-refractory ovarian cancer: results from the first 1,000 patients registered to National Cancer Institute Treatment Referral Center 9103.. J Clin Oncol.

[OCR_00420] Wani M. C., Taylor H. L., Wall M. E., Coggon P., McPhail A. T. (1971). Plant antitumor agents. VI. The isolation and structure of taxol, a novel antileukemic and antitumor agent from Taxus brevifolia.. J Am Chem Soc.

